# MMP-2—Potential Predictor of Epithelial–Mesenchymal Transition in Squamous Cell Carcinogenesis

**DOI:** 10.3390/life15071060

**Published:** 2025-07-02

**Authors:** Doinița Temelie-Olinici, Walther Bild, Laura Gheucă-Solovăstru, Mihaela Perțea, Daniela-Anicuța Leca, Bogdan-Vasile Grecu, Ioana-Alina Halip, Mădălina Mocanu, Ioana-Adriana Popescu, Adriana-Ionela Pătrașcu, Dan Vâță

**Affiliations:** 1 Department of Morpho-Functional Sciences II, “Grigore T. Popa” University of Medicine and Pharmacy, 700115 Iasi, Romania; doinita.p.olinici@umfiasi.ro (D.T.-O.); vasile-bogdan.grecu@umfiasi.ro (B.-V.G.); 2 Center of Anthropologic and Biomedical Research of the Romanian Academy Iasi, 700505 Iasi, Romania; 3 Department of Dermatology, “Grigore T. Popa” University of Medicine and Pharmacy, 700115 Iasi, Romania; solovastru.gheuca@umfiasi.ro (L.G.-S.); alinaioanahalip@gmail.com (I.-A.H.); madalina.mocanu@umfiasi.ro (M.M.); adriana.popescu@umfiasi.ro (I.-A.P.); patrascuai@yahoo.com (A.-I.P.); dan.vata@umfiasi.ro (D.V.); 4 Department Plastic Surgery and Reconstructive, Faculty of Medicine, “Grigore T. Popa” University of Medicine and Pharmacy, 700115 Iasi, Romania; mihaela.pertea@umfiasi.ro; 5 Department of Plastic Surgery and Reconstructive Microsurgery, “Sf. Spiridon” Emergency County Hospital, 700111 Iasi, Romania; 6 Department of Infectious Diseases (Internal Medicine II), Faculty of Medicine, “Grigore T. Popa” University of Medicine and Pharmacy, 700115 Iasi, Romania; daniela.leca@umfiasi.ro

**Keywords:** MMP-2, carcinogenesis, epithelial–mesenchymal transition, premalignant skin lesions

## Abstract

Epithelial–mesenchymal transition (EMT) is one of the key steps in cutaneous carcinogenesis. At the molecular level, this cellular dedifferentiation is modulated by the interaction of signalling pathways that favour basement membrane degradation under the influence of proinflammatory cytokines and matrix metalloproteinases (MMPs). Given the intricate role of these endopeptidases in modulating extracellular matrix turnover, the present study aimed primarily to identify the MMP-2 expression profile during the early stages of cutaneous malignant transformation. Forty-eight lesions with malignant transformation potential were excised in healthy tissue. Following the histopathological diagnosis of keratoacanthoma, Bowen’s disease and actinic keratosis, the biological preparations were deparaffinised and homogenised in order to perform the FRET technique using the “MMP-2 Assay Kit Fluorometric”. The results of the previous part of this research indicate that MMP-2 expression is more intense in lesions of actinic keratosis compared to normal tissues and to keratoacanthoma or Bowen’s disease lesions, inversely proportional to the histopathological degree of dysplasia. Monitoring metalloproteinase activity in dysplastic epithelium may improve the detection of malignant transformation and guide treatment decisions.

## 1. Introduction

Today, skin cancers represent some of the most frequently diagnosed epithelial neoplasms worldwide [1,2,3]. According to the NICE guidance on skin cancer, they account for 27% of all consultations and 1.7% of all skin lesions [2,3,4,5]. Among the Caucasian population, the most common cutaneous malignancies are non-melanocytic malignancies, represented by squamous cell carcinoma (20%) and basal cell carcinoma (80%), with their incidence increasing by 3–8% each year [1,2,4].

Multiple population-based studies show an increase in the incidence of cutaneous squamous cell carcinoma, both in areas with increased sun exposure near the equator and in areas with lower exposure, such as Finland, where the incidence has increased by 4% in recent decades. This tumour’s invasive and metastatic nature increases morbidity by 5% annually in Central Europe and contributes significantly to cancer-related deaths [4,5,6,7].

The risk of developing squamous cell carcinoma is mainly influenced by the presence of premalignant lesions or lesions with the potential for malignant transformation, such as actinic keratoses, keratoacanthomas and Bowen’s disease lesions [4,6,7,8,9,10]. With a prevalence ranging between 0.76 and 5% in Asia and 13.4% in the Americas, these cutaneous lesions exhibit cellular and molecular features whose description facilitates the identification of pathogenic mechanisms specific to the early stages of epidermal carcinogenesis [3,4,9].

In view of their increased risk of transformation into invasive cancers, *actinic keratoses* are considered by many clinicians and researchers squamous cell carcinomas in situ, which occur in about 75% of cases on photoexposed areas [1,2,3,4,5]. As an end result of the interplay of genetic and epigenetic factors, these cutaneous lesions are responsible for the clinical expression of approximately 60% of all squamous cell carcinomas. The risk of the malignant transformation of actinic keratoses is estimated to be 6–10%, with a variable prevalence depending on the histological type, the age group studied and the presence/absence of immunosuppression. The annual individual malignant degeneration of actinic keratoses ranges from 0.025 to 20% [1,2,3,4,5]. At present, precise quantification of the risk of malignant transformation is difficult. Given that the natural evolution of actinic keratoses typically involves high turnover rates, with lesions developing, regressing and recurring over time, one can explain why monitoring epithelial cell polarity may constitute an important pivot of the correct management of these photodermatoses [1,2,3,4,5].

Keratoacanthomas, epithelial tumours derived from pilosebaceous follicles, present a heterogeneous prognostic evolution marked by proliferation and invasiveness, regression and/or recurrence [6,7]. The risk of malignant transformation is approximately 15%. The clinical and histopathological features of keratoacanthomas and squamous cell carcinomas frequently overlap; therefore, a differential diagnosis is difficult to establish and quantify. Therefore, in the fourth edition (2018), compared to other editions, the World Health Organisation–International Agency for Research on Cancer (WHO-IARC) classified keratoacanthoma as a well-differentiated variant of squamous cell carcinoma. Histopathologically, malignant transformation is suggested both by the presence of squamous cells with atypical mitoses and hyperchromatic nuclei and by the loss of cell polarity [8,9,10,11,12].

Bowen’s disease or squamous cell carcinoma in situ is described as an intraepidermal, intraepithelial carcinoma with a risk of invasiveness of approximately 3–8% [4,5]. However, studies performed after 2017 reveal a risk of transformation to invasive squamous cell carcinoma of 16.3% [5,10].

It is well known that the morpho-clinical and prognostic evolution of a premalignant lesion, and implicitly of a malignant lesion, is conditioned by the cellular interactions that create the favourable tumour microenvironment. The dynamics of these interactions explain the mosaic of phenotypic changes associated with a variable spectrum of therapeutic and prognostic evolution. Moreover, these types of heterogeneous clinical expression are supported and propagated at the ultrastructural level by the constitution and persistence of specific molecular patterns [3,4,5,6,7,8,9].

The transition from dysplasia to invasive cancer follows the epithelial–mesenchymal transition (EMT) mechanisms, a key carcinogenesis step that helps tumour cells evade immune control. This cascade interacts with other molecular mechanisms and recruits a number of proinflammatory cytokines, such as interleukin-6 (IL-6) and tumour necrosis factor-α (TNFα), cytoskeletal remodelling and basement membrane degradation. Thus, through the intricate action of proinflammatory enzymatic and cytokine factors, cellular metabolism is influenced and directed towards the final proteolytic digestion response of the extracellular matrix, a key moment of epithelial–mesenchymal transition (EMT) [8,9,10].

### 1.1. Epithelial–Mesenchymal Transition

The morphological support of the cellular signalling cascade characteristic of carcinogenesis is provided by cell adhesion complexes. Thus, cell–cell and cell–extracellular matrix–cell junctions are responsible for the maintenance of normal tissue architecture [8,9]. In epithelial cells, they create and facilitate the functional interdependence of apical and basal structures, with a particular impact on terminal tissue differentiation. Transmission electron microscopy reveals the presence of a latero-apical junctional protein complex (zonula occludens, zonula adherens and desmosomes) with a pentalamellar appearance, formed by five parallel bands that are delimited by the homophilic interactions of adhesive molecules [13,14]. Therefore, the tight pentalamellar junctions recruit at the apical pole protein structures that sequentially and consecutively engage the basement membrane, a dynamic, highly specialised extracellular matrix structure, via cytoskeletal elements. The whole structure functions as an entity with apical–basolateral polarity capable of correctly integrating signals received from neighbouring cells. This morpho-functional characteristic distinguishes epithelial cells from neighbouring mesenchymal cells [10,11,12].

When the epithelial cell becomes vulnerable, a copying mechanism of mesenchymal cell behaviour is set up, a process known as epithelial–mesenchymal transition. As early as four decades ago, it was proposed and accepted that changes in EMT favour the initiation and development of carcinogenesis. Moreover, all epithelial cancers (about 80% of all oncological pathologies) are considered to have the activation of this dynamic process as a determinant step [10,12,15,16,17].

After the 1990s, there was an increase in the dynamism of studies focused on the identification and morphological and molecular quantification of EMT. Thus, among the working hypotheses resulting from this specialised research is the one arguing the link between embryonic EMT induced by various transcription factors and the progression of carcinogenesis. At the same time, the mechanisms of the transcriptional control of EMT have shown that the initiation and progression of EMT are reversible and independent of DNA sequence modifications [5,14,15,16].

In the identification and differentiation of epithelial and or mesenchymal phenotypes, the expression of the intermediate filaments of the cytoskeleton, proteins that stabilise the specialisations of the biological membrane, both at the lateral and basal poles, is involved. Thus, the type and level of cytokeratin expression are associated with the epithelial pattern, while vimentin functions as a marker of mesenchymal cells, cells lacking homotypic adhesiveness [17,18]. On the other hand, mesenchymal cells are able to express some extracellular matrix proteins, such as fibronectin and collagen fibres I and III. According to recent results, EMT constitutes one of the important sequences in the transformation of fixed, stable epithelial cells into mobile, invasive cells. In this context, it is demonstrated that the level of vimentin expression can be directly correlated in direct proportion to the metastatic risk of transformed epithelial cells in all epithelial malignancies [8,9,10,15].

Among the enzymatic regulators of this complex process, one can mention the matrix metalloproteinases, whose action tightly controls cell differentiation. Activation of this cascade involves glycoproteins, glycolipids and glycocalyx structural proteoglycans. In dynamics, the proteolytic activity is reflected in the latero-apical junctional protein complexes via cytoskeletal elements. Thus, the disorganisation of tight junctions favours the loss of polarity of epithelial cells induced by the disorganisation of the extracellular matrix under the action of metalloproteinases. All these molecular changes orchestrate the creation of an invasive and metastatic tumour microenvironment through the continuous secretion of activated metalloproteinases, which are able to evade the vigilance of the immune response [9,18].

The regulation of the activity of many metalloproteinases is performed by several transcription factors responsible for controlling the steps of epithelial–mesenchymal transition. Moreover, metalloproteinases are thought to represent the gene targets of epithelial–mesenchymal transition, and their expression constitutes the final event of EMT. However, the results from clinical studies have been modest and conflicting [12,17].

### 1.2. Subsection Metalloproteinase-2 (MMP-2)

In the etiopathogenesis of epidermal carcinogenesis, exposure to ultraviolet radiation is frequently blamed for inducing the expression of several target genes, including matrix metalloproteinases (MMPs). While their role in tumour formation remains debated, these zinc-dependent enzymes regulate extracellular matrix turnover and activate EMT-related pathways [10,11,12].

As early as 2015, research by Kessenbrock et al. correlated changes in the expression of metalloproteinases with phenotypic variability in cutaneous epitheliomas carcinomas [17]. The expression of these endopeptidases is associated with a heterogeneous array of physiological and pathological processes of active tissue remodelling. Through direct enzymatic action, they may make the extracellular matrix, the barrier to the invasiveness and metastasis of cutaneous tumour dermatoses, vulnerable. In addition, various metalloproteinases are secreted during tumour proliferation, invasion, metastasis and angiogenesis, affecting the tumour microenvironment through induced dynamic changes. Their effects are clinically translated into a worsening prognosis. They are expressed by both tumour cells and adjacent stroma [11,12,14,17,18,19,20].

Over the years, 30 matrix metalloproteinases that are involved in the homeostasis and remodelling of tissue architecture through the proteolysis of extracellular matrix components have been studied. They regulate the activity of other proteinases, growth factors, cytokines, chemokines and cellular receptors, with important roles in the progression of EMT [12,14,19].

Depending on the specificity of the degraded or cleaved protein substrate, several main groups of matrix metalloproteinases can be identified: collagenases—MMP-1, -8, and -13; gelatinases—MMP-2 and -9; stromelysins—MMP-3, -10, and -11; matrilysins—MMP-7 and -26 and membrane-type metalloproteinases—MT-MMP. Another classification system reports the existence of six main groups: **A**: MMP-19, -26 and -28; **B**: MMP-11, -21 and -23; **C**: MMMP-17 and -25; **D**: MMP-1, -3, -8, -10, -12, -13 and -27; **E**: MMP-14, -15, -16 and -24; and **F**: MMP-2, -7, -9 and -20 [11,12,15,16].

All metalloproteinases have three well-defined, stable domains in their structure: the “predomain” with a role in signalling to the endoplasmic reticulum, the “prodomain” responsible for maintaining the inactive form and a catalytic domain. Their interactions with specific sub-layers are mediated by the C-terminal hemopexin-like domains, domains linked by a flexible linker to the catalytic centre. With the exception of MT-MMPs, metalloproteinases are synthesised as inactive, latent zymogens and then secreted into the extracellular environment. At this level, they acquire their active, catalytic state in a controlled manner by prodomain removal. Secreted pro-metalloproteinases (pro-MMPs) can be activated in vitro with the help of plasmin, trypsin or kallikrein [11,16,21].

Pro-MMP-2 is activated by MT1-MMP, which, together with the tissue inhibitor of MMP-2 (TIMP-2), forms a receptor structure on the cell surface. TIMP-2 has a free C-terminal end that binds pro-MMP-2; thus, MT1-MMP released from binding to TIMP-2 can activate pro-MMP-2. Low levels of TIMP-2 stimulate the activation processes. In numerous normal physiological conditions, pro-MMP-2, similar to pro-MMP-9 and -12, can be activated by the uPa/plasmin system. Pro-MMP-2 can also form complexes with TIMP-4 and MMP-9 [11,15,20,21].

MMP-2, 72 kDa, is expressed by a wide variety of normal and transformed cells, including fibroblasts, keratinocytes, endothelial cells, chondrocytes and osteoblasts. Among all metalloproteinases, the gelatinase group, consisting of MMP-2 (gelatinase A) and MMP-9 (gelatinase B), is considered to play an important role in malignant tumour development due to their ability to degrade extracellular matrix components, including collagens types IV, V, VII, X, XI and XIV, elastin, proteoglycans and fibronectin [20,21,22].

Type IV collagen is the protein skeleton of the extracellular matrix and plays a very important role in maintaining the integrity of the basement membrane and protecting it from the harmful action of various biological, physical and chemical factors. It separates epithelial cells from the surrounding mesenchyme. Its degradation, in particular under the action of MMP-2, also called type IV A collagenase, affects the integrity of the basement membrane, favouring invasiveness, neovascularisation and metastasis, which are negative prognostic factors in all cancers. It has also been shown that this MMP-2 gelatinase is highly expressed during the early stages of squamous carcinogenesis and plays an important role in tumour initiation and growth [19,21,22,23].

Taking all these aspects into account, the main aim of the present study was to identify and correlate MMP-2 expression with the morpho-clinical and evolutionary phenotype of the most common cutaneous lesions with malignant transformation potential. Consequently, it can be seen how the evaluation of this enzyme biomarker can complete the bio–histological–molecular panel for the specific and complete diagnosis of cutaneous proliferating squamous cell lesions.

## 2. Materials and Methods

### 2.1. Study Design

In the present study, we selected skin fragments harvested by excisional biopsy with a scalpel blade from 48 female patients, who were non-smokers, without chronic alcohol consumption, aged 45–60 years and clinically and histopathologically diagnosed with actinic keratosis (24 cases), Bowen’s disease (12 cases) or keratoacanthoma (12 cases). Fragments of normal skin were obtained by the same harvesting technique from the perilesional areas to constitute the control group.

Prior to surgery, informed consent was obtained from each patient in accordance with the legislation in force. The anatomopathological diagnosis was established in the Pathological Anatomy and Prosectology Service of the Clinical Hospital C.F., Iasi, and the Pathological Anatomy Laboratory of “Dr. I. Czihac” Military Emergency Clinical Hospital, Iasi, according to protocol no. 2365/2023, which was adopted and approved by the Ethics Committee.

The present retrospective study established the analysis period as 1 January 2013–1 January 2023. Initially, the diagnosis was established according to the WHO criteria, 3rd edition. Subsequently, to eliminate any terminological ambiguity, the histopathological description was adapted according to the recommendations established by the WHO 4th edition (2018) (Table 1). Thus, in the second stage, the histological evaluation of tissue lesions redefined the term dysplasia from the first stage and focused on the type and degree of atypia, stromal invasion and the status of the resection margin.

Several fragments were obtained from the tissue samples collected from the patients included in the study, which were processed by the *paraffin-embedding* technique, after which some were sectioned and then stained with *haematoxylin–eosin* for anatomopathological diagnosis. Others were deparaffinised for use in the *FRET technique*.

### 2.2. Homogenisation of Samples

Tissue samples were pre-weighed and homogenised using an ultrasonic homogeniser (including those that required prior paraffin stripping): 20 s pulses at medium intensity in an extraction buffer (volume—1 mL, 10 mM Tris pH 7.4, 150 mM NaCl and 1% Triton X-100). During homogenisation, all samples were kept on ice and cooled between ultrasonication steps. The homogenates obtained were transferred into 1.5 mL Eppendorf tubes and centrifuged at 13,000 rpm for 10 min at 4·°C. The supernatants obtained were separated and stored at −20 °C during biochemical determinations.

### 2.3. Determination of Tissue Protein Levels

Protein determination was performed with the “BCA Protein Concentration Determination Kit” (Pierce™ BCA Protein Assay Kit 23225, produced by Thermo Fisher Scientific Inc., Waltham, MA, USA). The method is based on the interaction of Cu^2+^ ions with proteins in an alkaline medium, followed by the detection of Cu^1+^ copper ions with biquinic acid and determination of the final product by colourimetry. BSA (bovine serum albumin) was used as a standard. After making the working reagent (5 mL reagent A and 0.1 mL reagent B), 10 μL of the supernatant (or standard solution) was combined with 200 μL of the working reagent. After 30 min incubation at room temperature and cooling, the samples were read colourimetrically at 562 nm using a Picos/Belgium semi-automatic biochemistry analyser.

### 2.4. Determination of MMP-2

The determination of metalloproteinase-2 was performed using the FRET (Fluorescence Resonance Energy Transfer) technique with the help of a fluorometric kit “MMP—2 Assay Kit Fluorometric” (cod AS-60569, AnaSpec (Fremont, CA, USA), Bio-Techne (Minneapolis, MN, USA)). The artificial FRET peptide contains a fluorochrome 5-FAM (carboxyfluorescein) and QXL520 quencher QXL™ 520 (a fluorescence inhibitor). The substrate sequence is 5-FAM–Gly–Pro–Leu–Gly~Val–Arg–Gly–Lys–QXL520, and the specific cleavage occurs between Gly and Val residues, a site recognised by MMP-2. The cleavage of this peptide by MMP-2 (gelatinase A) results in the removal of the inhibitor and the appearance of a fluorescent signal that is proportional in time to the enzyme activity. The protocol used required the reconstitution and dilution of the kit buffers and reagents. The working reagent containing the FRET peptide (100 μL) was mixed with the samples, standards and controls (100 μL) in a 96-well plate, and the proteolytic activity was monitored in a Genios Tecan/Salzburg, Austria plate reader (fluorescence module, excitation filter 490 nm and emission filter 520 nm). The measured relative fluorescence units (RFU) were related to the amount of protein in the samples.

### 2.5. Statistical Processing

Derived indicators are intended to emphasise the qualitative aspects of a whole, aiming at the relationship between different parts of a group of patients or different characteristics and the interdependency links between variables. The following derived indicators were used: mean value indicators: simple arithmetic mean, median, and module; dispersion indicators: standard deviation and variance.

The Coefficient of Variation (CV%) emphasises the percentage deviation between two means, providing results on the homogeneity of the series of values, and the Skewness test (−2 < t < 2) validates the normality of the series of values. It is used when the examined variable has continuous values. The mean value close to the median and the result of the Skewness test in the range [−2; +2] suggest the homogeneity of the series of values and whether significance tests for continuous variables can be applied.

The F-test (ANOVA) was used when comparing 3 or more groups with normal distributions in conjunction with the Turkey correction (post-hoc Turkey) to reduce the error rate when testing several hypotheses.

## 3. Results

In patients with actinic keratosis-type lesions, MMP-2 varied in the range of 117–133 RFU/mg protein, with a variance of 17.74%, the median (124.50 RFU/mg protein) was close to the mean level of 124.46 ± 4.21 RFU/mg protein, and the result of the Skewness test (*t* = 0.076) suggests homogeneity of the value series (Table 2).

In patients with keratoacanthoma, the MMP-2 variations were in the range 85–102 RFU/mg protein, with a variance of 6.40%, the median (95.50 RFU/mg protein) was close to the mean level of 94.58 ± 6.05 RFU/mg protein, and the result of the Skewness test (*t* = −0.256) suggests the homogeneity of the series of values (Table 2).

For cases with Bowen’s disease lesions, MMP-2 varied in the range 70–81 RFU/mg protein, with a variance of 18.36%, the median (77.50 RFU/mg protein) was close to the mean level of 76.0 ± 4.29 RFU/mg protein, and the result of the Skewness test (*t* = −0.291) suggests homogeneity of the series of values (Table 2).

**Figure 1:**
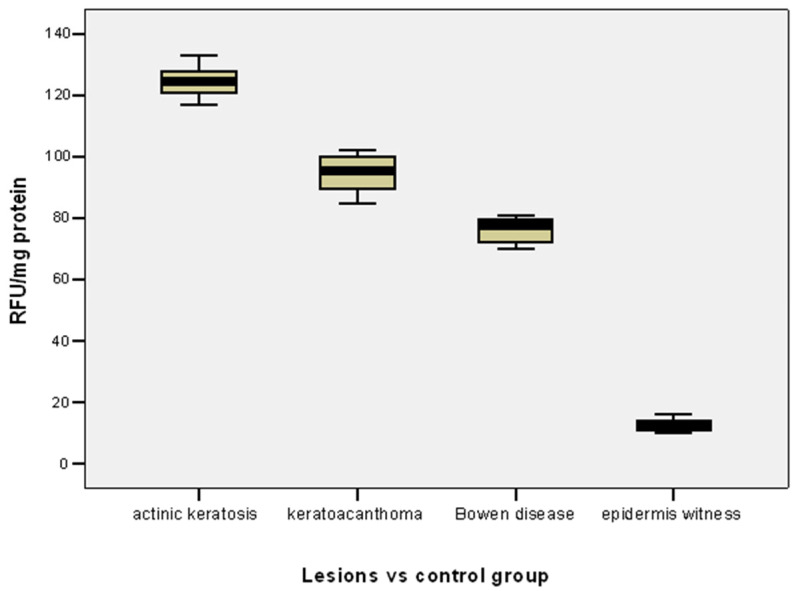
Comparison of MMP-2 expression between lesions and epidermis witness controls.

The mean level of MMP-2 was significantly higher in actinic keratosis lesions compared to keratoacanthoma (124.46 vs. 94.58 RFU/mg protein; *p* = 0.001) or Bowen’s disease lesions (124.46 vs. 76.0 RFU/mg protein; *p* = 0.001) (Table 2, Figure 1).

The mean level of MMP-2 was significantly higher in keratoacanthomas than in Bowen’s disease lesions (94.58 vs. 76.0 RFU/mg protein; *p* = 0.001) (Table 2, Figure 1).

The mean MMP-2 level was significantly lower in the control group (epidermis witness) compared to the mean MMP-2 level in both actinic keratosis lesions (12.70 vs. 124.46 RFU/mg protein; *p* = 0.001), keratoacanthoma (12.70 vs. 94.58 RFU/mg protein; *p* = 0.001) or Bowen’s disease lesions (12.40 vs. 76.0 RFU/mg protein; *p* = 0.001) (Table 2, Figure 1).

**Figure 2:**
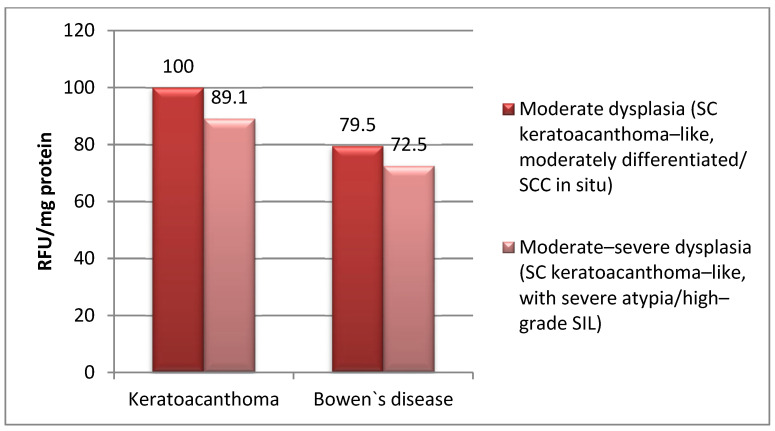
Comparison of MMP-2 expression according to severity of dysplasia (degree of atypia).

The mean level of MMP-2 was significantly lower in cases with moderate–severe dysplasia both in cases with keratoacanthoma (SC keratoacanthoma-like, moderately differentiated/with severe atypia) (100.0 vs. 89.17 RFU/mg protein; *p* = 0.001) and in cases with Bowen’s disease (SCC in situ/high-grade SIL) (79.5 vs. 72.5 RFU/mg protein; *p* = 0.001) (Table 3, Figure 2).

The mean level of MMP-2 was significantly lower in cases with Bowen’s disease than in cases with keratoacanthoma, both in cases with moderate dysplasia (SC keratoacanthoma-like, moderately differentiated/SCC in situ) (100.0 vs. 79.5 RFU /mg protein; *p* = 0.001) and in cases with moderate–severe dysplasia (SC keratoacanthoma-like, with severe atypia/high-grade SIL) (89.17 vs. 72.5 RFU/mg protein; *p* = 0.001) (Table 2, Figure 2).

## 4. Discussion

Squamous cell carcinoma, the second most severe cutaneous neoplasm, involves a multifactorial, genetic and epigenetic etiopathogenesis that synergistically activates the malignant proliferation of epidermal keratinocytes. In this way, the so-called cancerisation field is created [1,2,3,4,5,24]. The degree of invasiveness and the risk of metastasis are conditioned by the maintenance of the integrity of the extracellular matrix architecture and thus of the basement membrane. Therefore, their proteolytic degradation ultimately results in the anarchic remodelling of tissue structures via the continuous activation of aberrant cell signalling pathways [1,2,3,4,5,8,9,10].

The cascade of squamous cell carcinogenesis is maintained by complex cellular processes that mediate the sequential progression of normal tissue through a sequential spectrum of lesions, including hyperplasia, dysplasia, carcinoma in situ and invasive carcinoma. Therefore, the correct management of premalignant precursors and/or potentially malignant skin lesions is a key step in the correct diagnostic and therapeutic management of squamous cell carcinoma [2,3,4,5,10]. In this respect, three of the most important and common premalignant skin lesions were recruited and studied in the present study, namely, actinic keratoses, keratoacanthomas and Bowen’s disease (Table 2).

Interactions between neoplastic and normal cells have a significant influence on the progression of epithelial cancers [12,25]. In this context, the theory that the initiation and [19,26,27,28,29] progression of carcinogenesis is a consequence of impaired interactions between tumour cells and the surrounding tissue microenvironment is increasingly being addressed [19,30,31]. Experimental studies, carried out on 2D and 3D cell cultures, support the idea that neoplastic cells have the ability to correct their phenotype and behaviour when surrounded by a normal tissue microenvironment [19,26,27,28,29].

At the cutaneous level, dermo–epidermal interactions favour and control epidermis-specific cell growth and differentiation by correctly capturing and integrating the signal [19,26,27,28,29] processing of physicochemical and biological mediators, which is carried out by membrane junction complexes that define the apical–basal polarity specific to epithelial cells [25,26,27,30,31]. When this polarity is altered or lost, the cells transform, gradually accumulating mesenchymal cell characteristics in the so-called epithelial–mesenchymal transition [10,25].

Epithelial–mesenchymal transition is an important moment of structural definition for both the physiological mechanisms in embryogenesis and the pathological mechanisms found in inflammation and cancer. Thus, epithelial cells acquire the morphological characteristics of fibroblasts [10,17,18]. In recent decades, several studies have evidenced that epithelial–mesenchymal transition is the result of the interplay of several complex epigenetic control programmes. The key players that activate the respective interdependent mechanisms include proinflammatory cytokines, cytoskeletal elements and cell adhesion molecules, together with matrix metalloproteinases [27,28,29,32]. The expression of the latter is activated by ultraviolet radiation, an important exogenous stimulus for the development and transformation of premalignant and malignant skin lesion dermatoses. This favours the destruction of the extracellular matrix architecture, with notable effects on the increase in tumour invasiveness, angiogenesis and metastasis [32,33].

Taking into account the results of previous epidemiological studies, actinic keratoses, considered in situ neoplasms, are among the most common photo-induced dermatoses. Their invasive transformation is histopathologically documented by both the hyperplasia of atypical keratinocytes and the loss of cell polarity, changes that may ultimately result in epidermal waviness [12,25]. This theory of ultraviolet radiation–nuclear factor NFkb-metalloproteinase interdependence is also supported by the results of the present study, which reveals a higher level of tissue expression in actinic keratoses compared to keratoacanthoma lesions and Bowen’s disease (Table 2, Figure 1). At the same time, the observations raised by the results of experimental studies using other tissue types to illustrate the essential role of metalloproteinase-2 in tumour progression are also confirmed. Thus, increased levels of MMP-2 are associated with an aggressive tumour phenotype in carcinomas of the oesophagus, pancreas, prostate, lung and ovary [10,15,34].

In most cases, the activity of metalloproteinases is controlled extracellularly; they are secreted in an inactive, latent form, and upon removal of the prodomain, they acquire an active, catalytic state. MMP-1 and -28 and MT-MMP are activated intracellularly by furin-like proteases associated with the Golgi complex. Secreted pro-metalloproteinases (pro-MMPs) can be activated in vitro with the help of plasmin, trypsin or kallikrein. Compared to other metalloproteinases, MMP-2 expression is differentially regulated, thus indicating a unique role in cell–matrix interaction, including tumour invasion. In experimental studies, laboratory animals in which MMP-2 expression is inhibited show reductions in angiogenesis, tumour growth and metastatic capacity [35,36,37]. On the other hand, immunohistochemical studies performed in the last decade in a cutaneous context reveal increases in MMP-2 expression in tumour stroma and the parenchyma of invasive squamous cell carcinoma. This increase is superior to basal cell carcinoma [24,38,39,40]. The expression of this protein–enzyme is also increased in actinic keratoses, similar to the results obtained in this research using FRET at the tissue level, a technique more sensitive than immunohistochemistry [32,33].

Recent data support the view that MMP-2, together with MMP-9, is one of the most important metalloproteinases involved in the pathogenesis of cutaneous squamous cell carcinoma, significantly influencing both the early stages of malignant transformation and the mechanisms of differentiation and metastasis. MMP-9 is activated by MMP-2, the expression of which may be positively correlated with advanced age, alcohol consumption and smoking, despite the many controversies identified in the literature [27,39,41]. Based on these observations, the current study aimed to eliminate possible interference with different predisposing and/or favouring factors of malignant transformation; thus, only female patients aged 45–60 years, who were non-smokers and without chronic ethanol consumption, were recruited.

In the last two decades, some working hypotheses have been put forward indicating the key role played by MMP-2 and MMP-9 in initiating and promoting epithelial–mesenchymal transition by the ultrastructural disorganisation of the extracellular matrix. Furthermore, it has been observed that these gelatinases are able to activate and degrade, based on a “snowball” principle, other pericellular and even cytoskeletal components, with a particular impact on the establishment and modification of the normal, premalignant or malignant cell phenotype. If MMP-9 expression is controlled by inflammatory cells, that of MMP-2 is the attribute of endothelial, epithelial and fibroblastic cells [27,32,42]. Therefore, the primary objective of the present study was to highlight and compare the expression of MMP-2 in both normal and lesional skin from recruited premalignant dermatoses (Table 2, Figure 1).

MMP-2 was initially thought to be produced by epithelial cells, and its activation achieved by cleavage of 80 amino acids from the N-terminal portion of procollagenase IV. It is now suggested that tumour cells have docking sites for binding metalloproteinases secreted by stromal cells, thus emphasising epithelial–mesenchymal interaction. After being activated by the tumour cell-produced factor EMMPRIN, pro-MMP-2, secreted by fibroblasts, is thought to interact with the membrane metalloproteasemases (MT-MMPs) of neoplastic cells. MMP-2 can also interact with tumour and endothelial cells via the αVβ3 integrin [11,30,32]. These activation cascades between metalloproteinases achieve a complex regulatory network of tissue proteolysis [41,43,44]. The increase in MMP-2 in the lesions in the present study (Table 1, Figure 1) could indicate an alteration in MMP-9 expression based on the intricate activation mechanism of these proteinases.

Monitoring metalloproteinase activity in the dysplastic epithelium identified in actinic keratosis lesions is a pertinent approach in the more sensitive assessment of malignant transformation potential [18,41], and immunohistochemical studies do not always reflect the exact percentage of immunopositive cells, possibly also due to the fact that MMP-2 expression is regulated by numerous overlapping transcriptional, translational and post-translational mechanisms [30,34,38]. Therefore, the results of the present study are not in agreement with the results of immunohistochemical investigations that have indicated the role of MMP-2 in malignant transformation by directly correlating with the degree of dysplasia [11,15,38]. This is evidenced by the low expression obtained in the dysplastic lesions studied, which is inversely proportional to the severity of dysplasia (Table 3, Figure 2). However, the present results support the working hypothesis we revealed in our ultrastructural study published in 2022.

**Figure 3:**
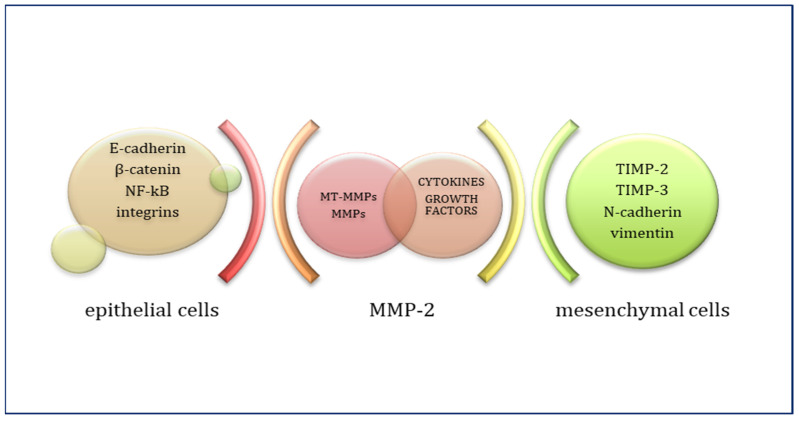
Molecular controllers of EMT.

In conjunction with the paraclinical data that formed the basis of the morpho-clinical reclassification of benign, premalignant and malignant skin lesions in the 4th edition of the WHO, the need to identify and characterise the interdependence of the numerous cellular and molecular factors that condition carcinogenesis is justified. For example, MMP-2 expression is controlled by its tissue inhibitor TIMP-2 (Figure 3). Pro-MMP-2 is activated by MT1-MMP, which, together with TIMP-2, forms a receptor structure on the cell surface. TIMP-2 has a free C-terminus that binds pro-MMP-2; thus, MT1-MMP released from TIMP-2 can activate pro-MMP-2. Low levels of TIMP-2 stimulate activation processes. Under numerous physiological conditions, pro-MMP-2, like pro-MMP-9 and -12, can be activated by the uPa/plasmin system. Pro-MMP-2 can form complexes with TIMP-4 and MMP-9. These activation cascades between metalloproteinases constitute a complex network for the regulation of tissue proteolysis [3,5,6,7,8,9,24].

On the other hand, given that most of the key events of cell differentiation and proliferation are concentrated at the level of the extracellular matrix, the intervention of Piezo channels (Piezo-1 and Piezo-2) can be recognised. This emerging hypothesis is at the intersection of tumour invasion biology, mechanotransduction and extracellular matrix remodelling. Some research indicates relevant functional links between MMP-2 and Piezo channels in carcinogenesis, invasiveness and metastasis. In this context, a directly proportional relationship of the expression of these channels with that of MMP-2 expression has been indicated [45,46].

The onset of EMT is also preceded by the ability of transformed epithelial cells to express stromal MMPs. MMP-2 is secreted by stromal cells, which explains its stimulatory role on invasiveness through tumour cell interactions with stromal cells. Under normal conditions, the absence of MMP-2 and TIMP-2 can be explained by low secretion, suggesting the reduced availability of pro-MMP-2, which is essential for MMP-2 activation. In pathological conditions, TIMP-2 levels are very important through their effects on tissue homeostasis. Both epithelial and stromal cells are sources of MMP-2 and TIMP-2. Thus, in dysplastic lesions, both are expressed in the basal and parabasal cells of the overlying epithelium and in lamina propria fibroblasts. The presence of MMP-2 expression in epithelial and connective tissue cells suggests synergism between the two enzymes. In addition, neoplastic cells may produce heterogeneous MMP-2. The secretion of this enzyme involves different regulatory mechanisms via cytokines and growth factors [30,41,42,43].

Multiple metalloproteinases exert their functions by degrading basement membranes and various matrix components, as well as by activating chemokines and growth factors. Thus, these enzymes have been shown to play a crucial role in mediating apoptosis, cell proliferation and differentiation, with a cumulative effect on malignant transformation and tumour angiogenesis [26,27,28,29,41]. This may also explain the much lower level of MMP-2 expression in the studied perilesional tissues (Table 1, Figure 1).

In peritumoural areas of epithelial cancers, the presence of MMP-2-secreting stromal cells is evident. Currently, studies correlating the level of stromal reactivity with the severity of tumour lesions are not well documented and supported. On the other hand, it should not be overlooked that the expression of MT1-MMP, the main activator of MMP-2, functions as an extracellular signal capable of stimulating and ensuring the pericellular proteolysis of basement membrane components. In addition, the crossregulation of the epithelial and mesenchymal metalloproteinases—MMP-2, MMP-13, MMP-12, and MMP-8—has been highlighted. It is thought that, in contrast to epithelial markers, such as plakoglobin or cytokeratins 18 and 19, mesenchymal markers, TIMP-2, TIMP-3, thrombospondin-1 and/or α1VI/α2I integrins may predict the invasive and metastatic phenotype of tumour cells. In this context, the synergistic role played by MT1-MMP co-expression has also been suggested [11,12,15,16,26,27,31,42].

The correlation of metalloproteinase expression with specific changes in the epithelial–mesenchymal transition is also explained by the involvement of the co-transcriptional mechanisms of β-catenin, an important protein factor in the maintenance of the architecture of intercellular adherens junctions. In this regard, in some squamous cell carcinoma cell lines, the EMT-specific phenotype was associated with the loss of E-cadherin and induction of vimentin and MMP-2. MMPs are also able to cleave E-cadherin. Those biochemical observations may explain the reduced expression of MMP-2 in keratoacanthoma lesions and Bowen’s disease. According to other studies, a plausible scenario of reduced MMP-2 expression would be to arrest cell–cell migration [10,38,39,40].

On the other hand, some in vitro studies emphasise the importance of the MT1-MMP/MMP-2 axis in the activation of pathways of this dynamic process. MT-MMP, a potent activator of MMP-2, shows the same expression pattern as that of MMP-2 and may condition tumour invasiveness [37]. Therefore, it can be explained why heterogeneous tumoral foci can be found in a carcinomatous lesion in which epithelial cells show different stages of mesenchymal-malignant transition [28,29,32,33,34,35,36].

## 5. Study Limitations

Studies conducted by Sarioğlu et al. in 2001 [47] and continued by Määttä et al. in 2010 [48] show that MMP-2 and TIMP-2 expression positively correlate with the degree of dysplasia of laryngeal and vulvar mucosal lesions, respectively. At the cutaneous level, TIMP-2 expression correlates with the degree of dysplasia and is higher in lesions with severe dysplasia compared to those associated with mild dysplasia or in perilesional skin. Therefore, one of the main limitations of this study is the lack of verification of this hypothesis by the quantification of TIMP-2 at the tissue level using the FRET technique.

Another limiting factor of the present study is the inability to determine the immune status of the patients from whom the lesional skin fragments were harvested. In this way, the framework predisposing and favouring epithelial–mesenchymal transition via MMP-2 could have been much more accurately completed.

It is important to identify and quantify by the ELISA technique the level of MMP-2 and MMP-9 and the main protein substrates of these endopeptidases. In addition, the expression of the adhesion molecules of the latero-apical junctional complexes should be assessed in order to understand the molecular context initiating the epithelial–mesenchymal transition in cancer carcinogenesis.

## 6. Conclusions

The interdependence of genetic mechanisms conditioned by a series of cellular and molecular factors activated by exogenous and endogenous variable conditions explains the uniqueness of the individual systems of carcinogenesis activation. Their primary end result is the induction of epithelial–mesenchymal transition. This is the reason why further clinical–paraclinical studies are needed to identify the particularities of the molecular control of various cell-signalling pathways.

The heterogeneity of changes induced by epithelial–mesenchymal transition, a key step in the initiation and progression of epithelial carcinogenesis, underpins the phenotypic variability of cutaneous squamous cell carcinoma at the cellular level. Therefore, the identification and quantification of the morphological and molecular mechanisms controlling this process is one of the main goals of dermato-oncological practitioners.

As an emergent of epithelial–mesenchymal transition, MMP-2 explains its important role in carcinogenesis. Therefore, one can understand the recommendation of its use as a potential target in the bio–histological–molecular diagnosis of premalignant and malignant dermatoses. On the other hand, the idea of using MMP-2 as a valid chemotherapeutic agent in the development of effective antineoplastic therapies can be supported.

## Figures and Tables

**Table 1:** Histological classification of lesions according to WHO.

Lesion Type	Classification According to WHO 3rd Edition [1,2,3,4,5,6]	Classification According to WHO 4th Edition (2018) [1,2,3,4,5,6]
Actinic keratosis	AK without dysplasia	AK (low-risk lesion, type I)
	AK with mild dysplasia	SIL low-grade (or AK type II)
	AK with moderate dysplasia AK with severe dysplasia	SIL low-grade (or AK type II–III) SIL high-grade/Carcinoma in situ
Keratoacanthoma	KA with mild dysplasia	SC keratoacanthoma-like, low degree of atypia
	KA with moderate dysplasia	SC keratoacanthoma-like, moderately differentiated
	KA with severe dysplasia	SC keratoacanthoma-like, with severe atypia (or in situ/invasive)
Bowen’s disease	BD with moderate dysplasia BD with severe dysplasia	SCC in situ SCC in situ/High-grade SIL
	Severe intraepidermal dysplasia	SCC

AK = actinic keratosis; KA = keratoacanthoma; BD = Bowen’s disease; SC = squamous carcinoma; SCC = squamous cell carcinoma.

**Table 2:** Descriptive statistics of MMP-2 (RFU/mg protein) expression.

MMP-2 Expression	N	Mean	Std. Deviation	Std. Error	95% Confidence Interval for Mean		Minimum	Maximum
					Lower Bound	Upper Bound		
Actinic keratosis	24	124.46	4.212	0.860	122.68	126.24	117	133
Keratoacanthoma	12	94.58	6.052	1.747	90.74	98.43	85	102
Bowen’s disease	12	76.00	4.285	1.237	73.28	78.72	70	81
Epidermis witness	10	12.70	2.058	0.651	11.23	14.17	10	16
Total	58	88.98	40.141	5.271	78.43	99.54	10	133

**Table 3:** Comparison of MMP-2 (RFU/mg protein) expression according to dysplasia.

MMP-2 Expression	Dysplasia/Degree of Atypia		F_ANOVA_ Test
	Moderate (SC Keratoacanthoma-Like, Moderately Differentiated/SCC In Situ) Mean ± SD (Limits)	Moderate–Severe (with Severe Atypia High-Grade SIL) Mean ± SD (Limits)	
Keratoacanthoma	100.0 ± 1.41 (98–102)	89.17 ± 2.86 (85–93)	*p* = 0.001
Bowen’s disease	79.50 ± 1.52 (77–81)	72.50 ± 2.95 (70–78)	*p* = 0.001
FANOVA test	*p* = 0.001	*p* = 0.001	

## Data Availability

The data presented in this study are available on request from the corresponding author.
